# Coordination of laboratory and diagnostic services during public health emergencies: a qualitative study

**DOI:** 10.1186/s12889-026-26422-4

**Published:** 2026-02-05

**Authors:** George Adjeisah Adjei, Herman Nuake Kofi Agboh, Grace Adjei Okai, Lily Yarney

**Affiliations:** 1 Catholic Health Service Trust, Ghana, P. O. Box 9712 Airport, Accra, Ghana; 2 University of Ghana Business School, P. O. Box LG 78, Legon, Accra, Ghana

**Keywords:** Laboratory and diagnostics, Emergency preparedness and response, Marburg virus disease

## Abstract

**Background:**

Diagnostics and laboratory testing are critical components of facilities’ systems for emergency response to infectious diseases. Yet, critical gaps exist in the testing and diagnostic capacities of faith-based health providers, particularly those in low and middle-income countries, limiting their response to health emergencies. Accordingly, the Coronavirus Disease 2019 (COVID-19) Strategic Preparedness and Response Plan for the World Health Organization African Region (1 February 2021–31 January 2022) was used to examine the capacity for laboratory and diagnostic services in a Christian Health Association of Ghana’s (CHAG) facility during the Marburg Virus outbreak in Ghana.

**Method:**

To examine the fifth pillar of the WHO COVID-19 SPRP-AFR (2021), 15 clinical and nonclinical health workers from a CHAG facility and Ghana Health Service (GHS) staff were interviewed. Thematic analysis was used to analyse the data.

**Findings:**

The CHAG facility relied extensively on external assistance from government of Ghana during MVD outbreak. The major challenges identified include equipment and human resource constraints, over-reliance on external entities for testing, and delays in sample collection and turnover time, among others.

**Conclusion:**

Given the recent disease outbreaks in Sub-Saharan Africa, the government of Ghana and owners of healthcare facilities in Ghana must start resourcing their facilities with the relevant structural and non-structural equipment in readiness for future disease outbreaks.

**Supplementary Information:**

The online version contains supplementary material available at 10.1186/s12889-026-26422-4.

## Background

The importance of diagnostic and laboratory services for patient care during health emergencies is well established [1–3]. In 2023 alone, more than 40 countries in Africa experienced over 168 public health emergencies, of which infectious diseases accounted for 90% and 75% related to zoonotic diseases [4]. Given this, the inadequacy of laboratory and diagnostic capacity of Health Systems in Africa [5], including limited testing capacity and delayed sample processing, can substantially constrain their response capacity during emergencies [6, 7]. According to the World Health Organization Regional Committee for Africa [8], 70% of health facilities in Africa lack the required laboratory equipment and reagents to perform basic diagnostic tests, with facilities located in rural areas most affected. The availability of diagnostics in basic primary care and advanced primary care facilities in ten low and middle-income countries was 19.1% and 49.2%, respectively [8]. Parallel to this report, in 2023, the Africa Centre for Disease Control and Prevention [9] found 85% of African countries’ responses on laboratory supplies, including PCR reagents, extraction kits, and consumables, to be inconsistent. In addition, inadequate infrastructure (45%), limited government funding (43%), inadequate equipment management (35%), and inadequate human resources (23%) were reported [9].

Similarly, Ghana lacks an adequate network of health delivery laboratories to address diagnostic and testing needs during health emergencies [10]. The current health delivery laboratories, including the National Public Health Reference Laboratory (NPHRL), three Public Health Laboratories at Kumasi, Sekondi-Takoradi and Tamale, and two internationally recognized laboratories, namely Noguchi Memorial Institute for Medical Research (NMIMR), and the University of Ghana and Kumasi Centre for Collaborative Research (KCCR), among others, lack adequate financial, logistical and human resource to run optimally [10].

It is worth mentioning that, although faith-based healthcare facilities are major healthcare providers in low and middle-income countries, they are more deficient in their diagnostic and laboratory services than public healthcare providers [11]. In Ghana’s health system, for instance, faith-based health institutions account for at least 40% of public health service delivery [12]. According to Boulenger, et al. [11], faith-based health delivery organizations (FBOs) are highly strained by human, financial and logistical resources when responding to infectious disease outbreaks [13]. This weak institutional framework, insufficient resources, and inadequate disease surveillance mechanisms, including the lack of well-resourced and functional laboratory and diagnostic systems, render FBOs vulnerable to disease resurgence [14, 15].

On July 7, 2022, the Marburg Virus Disease (MVD) outbreak was declared in one of Ghana’s faith-based healthcare facilities, providing evidence on FBOs’ capacity to independently handle major health emergencies [13, 16, 17]. Despite receiving extensive support from the Ministry of Health in collaboration with other partners for the enforcement of infection prevention and control measures, criticisms emerged regarding the functionality of laboratory and diagnostic services, raising questions about the faith-based facility’s emergency preparedness and response (EPR) capacity without external support [18, 19]. Beyond public criticisms, however, the preliminary literature review on the subject revealed the lack of empirical evidence on laboratory and diagnostic services of faith-based health facilities in Ghana [16], limiting the adoption of relevant positionality on the subject to bolster FBOs’ readiness for future health emergencies [11, 16]. Given this knowledge and capacity gaps in the face of the significant role that faith-based healthcare facilities play in Ghana, the country stands a great risk of suffering catastrophic consequences in the event of major disease outbreaks in the future.

Accordingly, this research examined the impacts of laboratory and diagnostic activities on preparedness and response efforts during MVD outbreaks in Ghana [15, 16, 20]. The WHO COVID-19 Disease Strategic Preparedness and Response Plan for the African Region was used to examine reported views on laboratory infrastructure and capacity, specimen collection and transport, PCR testing turnaround times, biosafety and quality-assurance practices, and the availability of testing and protective equipment [14].

## Analytical framework

### WHO Coronavirus disease 2019 (COVID-19) Strategic Preparedness and Response Plan for the African Region (1 February 2021–31 January 2022)

SPRP-AFR (2022) is a regional framework against COVID-19 pandemic at the sub-national, national, and regional levels [14]. It emphasizes sustained inter-agency responses, allocating adequate resources, and enhancing the capacity of African nations to manage transmission, and mitigate adverse effects on families, economies, and health systems. The WHO COVID-19 SPRP-AFR (2022) comprises a matrix of eleven pillars, covering Coordination, Planning, Financing, and Monitoring (CPFM), Risk Communication, Community Engagement, and Infodemic Management (RCCE-I), Surveillance, Outbreak Investigation, and Calibration of Public Health and Social Measures (SOPHS), Points of Entry, International Travel, and Transport, and Mass Gatherings (PIM), Laboratories, and Diagnostics (LD), Infection Prevention and Control and Protection of the Health Workforce (IPCP), Case Management, Clinical Operations, and Therapeutics (CMCT), Operational Support, and Logistics, and Supply Chains (OLS), Strengthening Essential Health Services and Systems (SEHSS), Vaccination (V), and Research, Innovation, and Evidence (RIE) [14].

Although recent, there is a popular adoption of the tool by researchers to evaluate emergency coordination of healthcare units across the globe. For instance, Ngoy et al. [15] used it to investigate the “Coordination mechanisms for COVID-19 in the WHO Regional Office for Africa” and found that at least 87.23% of coordination strategies were implemented as a single layer or in conjunction with other coordination mechanisms. Similarly, in a scoping review on “Urban health nexus with coronavirus disease 2019 (COVID-19) preparedness and response in Africa,” Alhasan et al. [21] found that at least 27% of the articles focused on preparedness towards COVID-19. Both studies found the model to be a “one-size-fits-all” approach, for an effective health system response to emergencies.

This study is centred on the Fifth pillar of the SPRP-AFR (2022) framework, namely Laboratories and Diagnostics (LD), to assess the coordination of laboratory and testing systems during the outbreak of MVD in Ghana. Limiting the scope to this pillar enabled the critical analysis of laboratory operations during the outbreak, offering valuable insights against future huddles.

## Method

The research used the Coronavirus Disease 2019 (COVID-19) Strategic Preparedness and Response Plan for the WHO African Region (1 February 2021–31 January 2022) (SPRP-AFR) to guide data collection and analyses [14, 22]. A phenomenological qualitative design was utilized, anchored in Pillar Five (Laboratories and Diagnostics) of the SPRP-AFR framework. The study followed the Consolidated criteria for Reporting Qualitative studies (COREQ).

### Selection of participants and data collection

The study was located in one of Christian Health Association of Ghana’s (CHAG) primary hospitals. CHAG, the second largest health service provider in Ghana, operates 374 facilities with 34,589 employees delivering healthcare services at primary, secondary, and tertiary levels, reaching around 11,308,640 Ghanaians [12]. The referenced facility serves as the sole referral hospital in its administrative district. In 2022, it recorded 26,972 OPD attendances, with a bed capacity of 41, available bed days of 14,965, and 7,775 annual inpatient days. In addition to internally generated funds, CHAG facilities often rely on Ghana Health Service (GHS) to meet some of their operational costs. The study targeted clinical and nonclinical healthcare professionals actively involved in managing the MVD outbreak in the CHAG facility where the virus was detected. Participants were selected through purposive sampling, aligning with the research question. Authorization was obtained from CHAG, and participants invited via phone calls for interviews. A total of 15 participants were interviewed using a semi-structured guide focusing on pillar five of the WHO COVID-19 SPRP 2022. The tool was tested on three health workers with frontline experience during COVID-19 pandemic. The participants encompassed various roles, including District Deputy Chief Disease Control Officer, District SNO Public Health, Hospital Administrator, Senior Health Service Administrator, Senior Medical officer, Medical Director, Medical Doctor, Nurse Manager, Nursing Officers, Enrolled Nurse, Biomedical Scientist, and Human Resource Manager. The guide included sections for participants’ demographic characteristics, and the LD of the WHO COVID-19 SPRP 2021. The interviews were conducted virtually via Zoom in English from April 2023 to August 2023, recorded, transcribed, cleaned, coded, and analyzed using NVivo (version 14) [22]. To minimize external influences, participants were either required to isolate themselves during interviews or participate in the interviews after work hours when isolation was not feasible. The interviews lasted 20–45 min, and the number of interviews conducted, determined by data saturation [22].

### Data analysis

We applied Braun and Clarke’s thematic analysis for data thematization and analysis. The coding process involved six levels: organizing responses under six thematic areas of the 5th pillar of WHO COVID-19 SPRP 2022; summarizing similar ideas into subthemes aligned with WHO COVID-19 SPRP 2021; and deducing specific information from data to assess the institution’s response to MVD outbreaks in Ghana.

### Ethical considerations

Ethical approval was secured from the Ghana Health Service Institutional Review Board under review number GHS-ERC 007/02/23. All identifiers, including facility names and locations, were either substituted with pseudonyms or omitted from the documentation to ensure confidentiality. The study design followed the COREQ checklist for qualitative research.

### Reflexivity

The researchers had conducted research with CHAG in the past. This position provided contextual understanding of the institutional systems and coordination structures being studied. Given this, regular team discussions and validation of emerging themes were carried out to ensure that personal or institutional perspectives did not influence data analysis. Credibility was established through triangulation and participant verification of findings. Flexibility in interview timing and interview location enabled participants to speak freely, enhancing data accuracy.

## Findings

Table 1 presents the demographic characteristics of study participants. They comprise a District Deputy Chief Disease Control Officer, a Hospital Administrator, a Senior Health Service Administrator, a nurse manager, an enrolled nurse, a District SNO Public Health, a Senior Medical officer, a Medical Director, a Medical Doctor, 4 nursing officers, one biomedical scientist, and a human resource manager. Females (*n* = 9) outnumbered males (*n* = 6), with majority of them within 30–39 age range (*n* = 9), followed by the 40–49 age group (*n* = 4). The mean age of participants was 37 years (± 5 years), whereas the mean score for years of employment was 7 years (± 4.05 years). Eleven participants were married, 4 were unmarried, while 12 were parents. 

**Table 1:** Participant list

NAme	Designation	Age (years)	Gender	Marital status	Parental status	Years of employment
DDCO	District Deputy Chief Disease Control Officer	44	M	Married	Yes	2
DPHN	District SNO, Public Health	36	F	Married	Yes	5
HA	Hospital Administrator	43	M	Married	Yes	14
SHSA	Senior Health Service Administrator	34	F	Married	Yes	5
SMO	Senior Medical Officer	37	F	Married	Yes	4
MDir	Medical Director	42	M	Married	Yes	16
MD	Medical Doctor	46	M	Married	Yes	10
NM	Nurse Manager	37	F	Married	No	4
NO.1	Nurses Officer	29	F	Single	Yes	7
NO.2		36	F	Single	Yes	10
NO.3		34	F	Married	Yes	8
BO 4		39	F	Married	Yes	8
EN	Enrolled Nurse	26	F	Single	No	3
BS	Biomedical Scientist	31	M	Single	No	4
HRM	Human Resource	38	M	Married	Yes	5

Source: Field data, 2023

Table 2 highlights the main themes and subthemes from the analysis conducted.

**Table 2:** Emerging themes

Research objective	Themes	Sub-themes
Examine the coordination of laboratory and testing mechanisms in a faith-based healthcare facility in response to the MVD outbreak in Ghana	LD	- Laboratory Infrastructure and Capacity - Sample Collection and Transport - Testing Processes and Turnaround Times - Biosafety and Quality Assurance - Supply Availability (Testing Kits, PPE) - Surge Capacity and Staff Coordination

Source: Field data, 2023

### Laboratory infrastructure and capacity

Functional labs with required equipment, space, systems, and trained human resources are essential for enhanced diagnostic capability in facilities. Well-resourced labs ensure reduced reliance on external assistance during health emergencies and strengthen local preparedness and response to disease outbreaks. The nature of lab infrastructure, for instance, may determine the reliability of results, the use of prescribed processes for sample collection, compliance with biosafety standards, and the timeliness and accuracy of test results produced. The research explored participants’ views on the resourcefulness of the laboratory infrastructure during the outbreak of MVD in Ghana. Most of the participants who shared their opinions on this subject raised concerns regarding critical gaps in the capacity of the facility to diagnose and test cases during major outbreaks. Some of the participants had these to say:

*Infrastructure for the lab would also be appreciated. When a team came for research purposes*,* they were surprised that we could pick samples*,* store them*,* and transport them from such a small lab. … At any given time*,* can you only have one person in there? … Yes. (SHSA)*

*We called the disease control officer*,* he said he will come the next day and pick the sample to Noguchi. The next day he came and we did the necessary arrangement for the sample to be taken to Accra (NM).*

*At first*,* Noguchi said the first case was positive. But later*,* they said the sample from the second case wasn’t enough so we should bring another sample. But by then the dece*ased *had been deposited at a morgue and frozen so we couldn’t pick the sample. So they just came out* and said *that it is negative (DPHN).*

*We do not have enough staff at the lab. We run shifts. Some run morning shifts. We have for instance about five staff on duty but then in terms of the sampling area its one patient and one lab staff at a time. You can’t have for instance two lab staff at a time taking patients samples at a time. We were able to have at least four or five at a go but at the moment we need to be careful not to crash into their lab net (BS).*

*The lab has a fridge to store samples. So they kept the samples in the fridge*,* wrap it and they put it in a zip lock bag. They label the name*,* age of the person and what is necessary for identification. Anyone picking it up would likewise go with the vaccine carrier ice packs. The challenge occurs when they are taking the samples. We kept the initial samples for 3 days before sending to Accra. In Accra too*,* it delayed for about two weeks before we receive the feedback from Noguchi. The samples were also sent to Dakar for confirmation. The duration between the dispatch of the swap through the district directorate and the feedback was 2 weeks. Relying on the district directorate to take samples from the lab is challenging. … There are some samples sitting at the lab that the directorate is not coming for*,* after the whole incident*,* we were told WHO said Ghana is Marburg free so they never came for the rest of the samples. If we were mandated or having access to transfer the samples ourselves*,* we would have taken a lot more to Noguchi (DDCO).*

*Very bad*,* because we converted a room into an isolation room. If we have cases like that*,* we manage them in that room. Like I said*,* we even have to bring the monitors and other things into that room. (MD).*

### Sample collection & transport

The study explored the systems and protocols used by the facility to collect, pack, and transport samples and patients to ensure safety and quick diagnosis of diseases. A stable, reliable, and fast transportation network ensured the preservation of the integrity of samples for accurate and timely diagnosis and enhanced case tracking. When asked about the sample collection and transportation systems, some of them had this to say:

A *sample would be collected the following day … as there was a suspicion of Ebola. … About a week later*,* we received confirmation from the lab that it was*,* in fact*,* Marburg Virus.* *(NM)*

*For the first case … five days afterward [after sample collection]*,* I think… It took over six days for the results to return….We took the sample and handed it over to the disease control officer and the district health directorate for follow-up. … For this case … samples had to go to Noguchi instead of KCCR*,* and securing the funds required coordinating with the regional health directorate. (MDir)*

*I know that we informed the District Health Directorate when we picked the samples*,* and they were the ones who sent them to Noguchi. When the results came back*,* they reached the district level first*,* and they informed us. Any PPE we needed was also provided through the District Health Directorate*,* so we had good collaboration with them. Contact tracing for the first family was challenging. I remember when we went to collect samples from the community*,* it was difficult to reach them because I believe they had left the area and gone back to their hometown. So*,* from the information I gathered*,* it was quite difficult to trace them. Although we might have four or five staff on duty during morning shifts*,* we have to be careful not to crowd the lab*,* as it limits movement. (BS)*

*One challenge I noticed was with sample collection. We had to rely on the district directorate to take samples from the lab. I remember a conversation with the nurse manager*,* who expressed frustration that some samples were just sitting in the lab*,* and the directorate wasn’t coming for them. Later*,* we were told by WHO that Ghana is Marburg-free*,* so they never came for those samples. But if we were mandated or had access to take the samples ourselves*,* I know we would have taken a lot more. Sometimes*,* you have to call repeatedly before the disease control officer comes to pick up the samples. Recently*,* when we went for our performance review at the district*,* this issue was discussed thoroughly*,* and going forward*,* they promised to improve. I would say it has been somewhat addressed at the performance review meeting…We … designated one room for isolation. The IPC team inspected the area*,* performed the necessary decontamination*,* and conducted refresher training… We sent them home and management organized provisions for them to reduce the need to go out. (SHSA)*

*One other case came up from Bekwai. Initially*,* Noguchi Institute said it was positive*,* but later they mentioned that the sample brought to them wasn’t sufficient*,* so they asked us to provide another sample. By that time*,* the deceased had already been sent to a morgue and frozen*,* so we couldn’t retrieve another sample. (DPHN)*

### Testing processes & turnaround times

Sample contamination may occur as a result of the duration and processes used to test samples. For accelerated contact tracing, isolation, and quick clinical and public health decisions to be taken during health emergencies, short turnaround times are required. Given this, the participants’ views on the turnaround time for sample collection and testing were explored. Most of them raised concerns about the long turnaround time due to the distance between the facility and the testing centre, and the changing of test results.

*Initially*,* we suspected Ebola … we followed standard infection and control practices. … About seven days after the samples were collected*,* the district director called and informed us that the cases had tested positive. … We then proceeded with full isolation. (MDir)*

*From an IPC perspective*,* no*,* we’re not adequately prepared for outbreaks. PPE and equipment. Initially*,* they were just going in with standard equipment without knowing there was a Marburg case. After we confirmed it*,* they were required to use additional protective gear. (BS)*

*Initially*,* results showed two positive cases. Noguchi then sent samples to Dakar for further testing. When Dakar’s came back negative… People started questioning Noguchi’s findings. (SHSA)*

*I took two samples with me—one from our district and one from another district*,* Asante Bekwai District*,* which is a municipality. Since the CHAG hospital is located here*,* we included that sample because the person also showed similar unusual signs and symptoms. When we arrived at Noguchi*,* we submitted two samples: one from Ankaasi and one from here. Initially*,* the results showed two positive cases. However*,* after conducting their investigation*,* Noguchi decided to send the samples to Dakar for further testing. When Noguchi’s results came back positive and Dakar’s came back negative*,* it created some controversy. People started questioning Noguchi’s findings and favoring Dakar’s results*,* questioning the reliability of Noguchi’s tests. You understand what I mean*,* right? It was frustrating because Noguchi was assigned to conduct the investigation and provided positive results. But in the end*,* people seemed to believe Dakar’s results instead…when the results came back from Dakar*,* the sample from the facility (name deleted) was positive*,* but the sample from the other facility (name deleted) came back as negative. They explained that the sample from the other facility (name deleted) was insufficient to conduct a thorough investigation. (DDCO)*

*So the lab man took the sample and reported to the director. So they took the sample and called the directorate and he also called the regional office to inform them about the suspected case. So*,* the sample was taken to Noguchi and the result came out being positive for Marburg. (DPHN).*

### Surge capacity & staff coordination

The capacity to mobilise additional workers and resources during health emergencies is necessary to deal with high caseloads and prevent systems collapse during disease outbreaks. During the interviews, participants were asked to describe the types and capacity of service delivery networks available in the facility and the availability of emergency medical teams to respond to the outbreak. The result shows that there were emergency response teams available, yet their capacity to respond to major outbreaks was limited by the lack of requisite medical equipment and personnel.

*We had a short workshop on how to wear PPEs. But we lacked staff during that time because we were 15 staff for the unit*,* seven of us went to quarantine*,* so the nurse manager had to go and bring staff from other units to help at emergency unit (NO 2).*

*Contact tracing was a joint effort between our facility and the district. … Staff were categorized into risk groups: highest-risk (doctors/PAs)*,* medium-risk (nurses)*,* lower-risk (other emergency staff). Only first two groups were screened. (DDCO).*

*Taking the blood sample and following up was spearheaded by the district disease unit. They came to take the samples. During the first case*,* there was a blood splash on one of our nurses. Since it took two weeks for MVD to be confirmed*,* the staff was rushed to the emergency ward for monitoring after two weeks of the incident (SMO).*

*During the Marburg outbreak*,* the KCCR (Kumasi Centre for Collaborative Research) sent some representatives*,* and there were others from the GHS surveillance unit in Accra. The district health directorate coordinated the public education efforts with the district assembly. However*,* one challenge we encountered is that the district health management team (DHMT) does not include representatives from the hospital. This means there’s no doctor on the DHMT*,* so decisions are sometimes made without our input*,* and we later have to adjust to their plans. (MDir)*

*They organized a workshop on handling such cases. I think the Infection Control and Prevention (IPC) team came around to train us on the items we might need. (NO 3).*

*They directed us to wear PPEs whenever we were in the ward*,* especially near suspected cases. They converted one of the rooms in the emergency department into an isolation area to minimize exposure…We had basic supplies like gloves and masks*,* but not enough full protective suits. I remember feeling worried because we had to reuse some PPEs due to shortages*,* which wasn’t ideal. Reusing PPE doesn’t fully protect us*,* and it made me feel more exposed. (NO 4).*

### Biosafety & quality assurance

To prevent lab-acquired infections, the study explored the protocols enforced and the infrastructure available to ensure a safe environment and reduce lab transmission risks. Quality assurance was also explored to ascertain the degree of trust and reliability the public can place in test results during health emergencies.

*When transporting [samples]*,* we used a vaccine carrier with ice packs. From storage to transport*,* the handling process was secure. (SHSA)*

*They provided us with coveralls and gave us proper training on how to put them on and remove them safely. … All staff involved in care were quarantined for 14 days. (NM)*

*The PPEs were not enough for all staff*,* and there aren’t enough staff members in the emergency unit. (NO 2).*

*The first measure was to quarantine any staff who had direct contact with the patient. I also doubled as the IPC (Infection Prevention and Control) focal person for the facility. Within about three days of getting the news that the samples we sent had tested positive for Marburg*,* we quickly organized a training session for all the staff on standard and transmission-based precautions under IPC. We didn’t have specific training tools for Marburg*,* so we focused on standard and transmission-based precautions. We covered all 12 standard precautions*,* including hand hygiene*,* respiratory hygiene*,* and everything else under standard precautions. Since we knew Marburg was transmitted through contact*,* we placed extra emphasis on contact and transmission precautions. We went back to some of the measures we used during COVID*,* like spacing out patients for social distancing*,* especially in the OPD (Outpatient Department) and vital signs area…In routine situations*,* the cleaning staff usually wear basic gloves*,* aprons*,* and occasionally face masks when dealing with high-risk areas. However*,* in the case of an outbreak like Marburg*,* they needed to wear full PPE*,* including gowns*,* face shields*,* masks*,* and double gloves. Initially*,* they weren’t fully aware of the risk and went in with just routine protective gear. After we realized the seriousness of the situation*,* we upgraded their equipment and gave them specific training on handling high-risk cleaning*,* especially for waste management from isolation areas. The emergency unit has its own cleaning equipment. (BS).*

*The nurse manager took the lead in training the nurses. She invited the disease control team and the medical director also contributed to the training. We made sure that everyone received the necessary guidance while keeping gatherings small to minimize any risks. (HR).*

### Supply availability

Safety of frontline staff is a necessary requirement during health emergencies. The availability of necessary equipment, including diagnostic reagents, testing kits, and personal protective equipment, prevents infections and limits the accumulation of backlogs, enabling uninterrupted testing. When interviewed, majority of the participants complained about the inability of the lab in the health facility to perform tests. Others mentioned the insufficiency of testing kits and other protective equipment during the outbreak. Some of them had this to say:

*For cases like Marburg*,* our lab isn’t able to detect the virus directly. What we do is store the samples in our fridge*,* and when necessary*,* they are taken to Accra for testing. … It worked smoothly. We had no challenges with this process. (EN)*

*We didn’t have enough testing kits. Even the kits we did receive arrived late—they didn’t come at the right time. We had to wait*,* which was challenging. (SMO)*

*We also need an X-ray machine*,* which would be very useful in detecting cases involving respiratory tract infections. But most importantly*,* we need a proper isolation unit. Having an adequate and dedicated isolation unit would make a huge difference in how we manage infectious cases. (NM)*

*The district director supplied us with personal protective equipment (PPE). … They provided us with coveralls and training.* ***(EN)***

*Sometimes that personal protective equipment (PPE)*,* isolation centers*,* and even instruments become a challenge. Sometimes*,* we have to use tools or instruments like blood pressure (BP) apparatus in ways that aren’t ideal. Ideally*,* we should have an isolation room equipped with monitors*,* BP apparatus*,* and other instruments specifically for that room*,* but we don’t have these things. The truth is that we don’t receive much from them [District Health Directorate]. It was during the Marburg issue that they planned to bring us PPEs*,* but even then*,* it wasn’t much. (MD).*

*For example*,* if we have ten staff members in the emergency unit but only two or three gowns*,* it means we would have to select only certain people to go to the isolation ward. So*,* it wasn’t enough. And considering these items are disposable*,* it felt like we had to reuse them*,* even though that shouldn’t be the case. If I wear one*,* I shouldn’t have to pass it to someone else for next time. This approach could compromise infection control. Also*,* disinfecting the body suits became an issue since we didn’t have enough. I found that to be a major challenge. (NO 3).*

### Word cloud

**Fig. 1:**
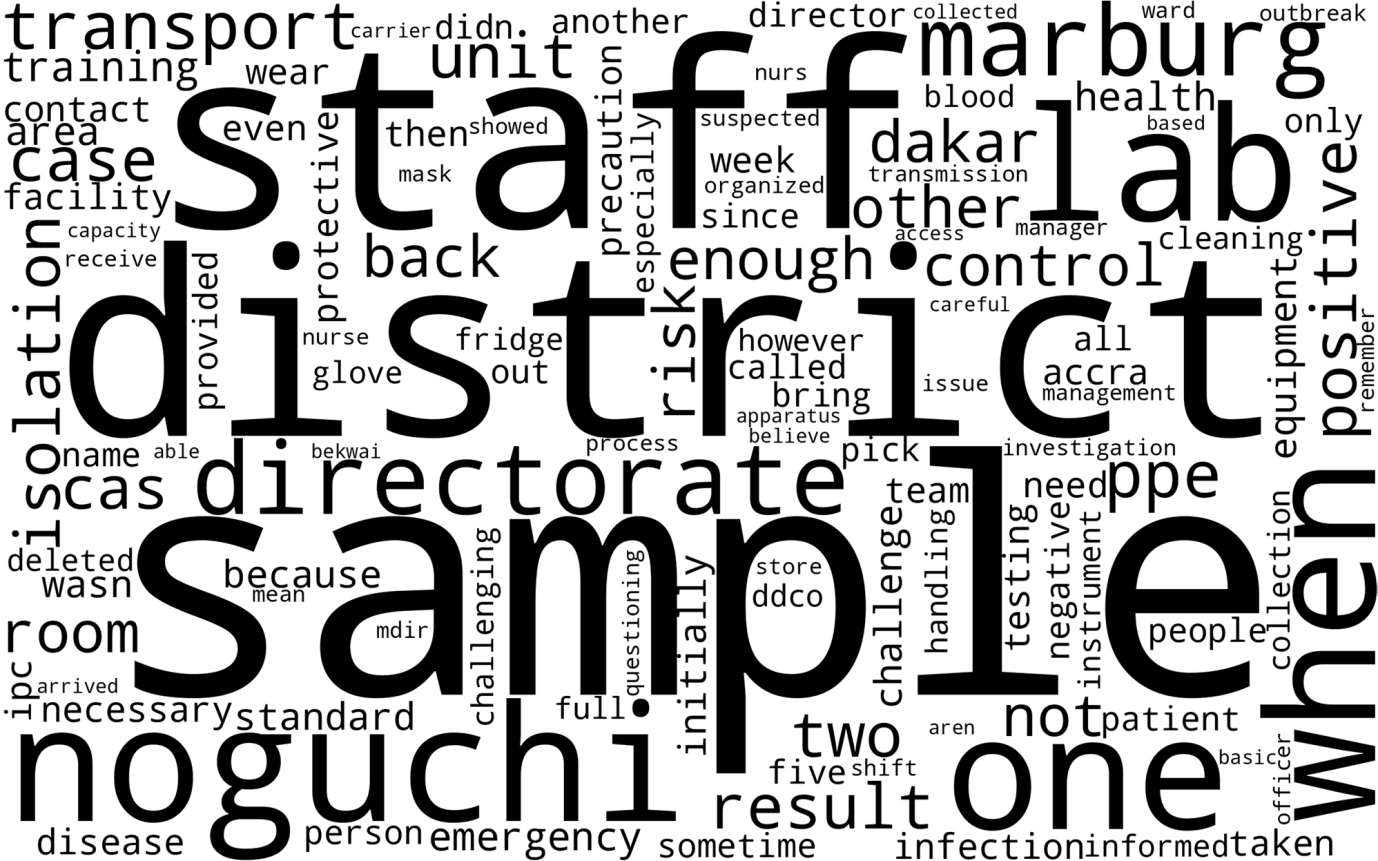
Word cloud. *Source: Field Data*,* 2023*

Figure 1 is a Word Cloud, derived from participants’ responses to questions on lab and diagnosis. The words used suggest the operational realities of officers working in or interfacing with laboratory and diagnostic processes. This excludes names of individuals and institutional structures, and generic words like “is, to, and, for, and by”, among others. The dominant terms observed were “samples, lab, staff, testing, fridge, transport and results”. The term “sample” also relating to testing, was the most frequently used, showing the significance of accurate and timely diagnostics, and the negative effects of delayed results and repeated sampling during the outbreak of lab operations. The terms “staff and coordination” were the next most frequently used, highlighting the human resource challenges faced by the facility during the outbreak, and the significance of inter-agency collaborations to ensure biosafety and reliable diagnosis during health emergencies. Finally, the terms “fridge and transport” occurred many times, showing the criticalness of health infrastructure for the preservation of samples, timely movement of samples to reference labs, and ensuring biosafety. Overall, the Word cloud highlights critical areas that must be strengthened.

## Discussion

The importance of well-resourced laboratories, particularly in rural and hard-to-reach areas, with appropriate equipment and biosafety measures was well established by participants of this research [14]. According to the WHO COVID-19 Strategic Preparedness and Response Plan (SPRP) 2021 operational guidelines, their effectiveness depends on compliance with the checklist for health laboratories and diagnostics during health emergencies. This includes laboratory workforce, lab supplies like cold chain, reagents and consumables, a network of laboratories and partners, testing, and distribution, among others. Despite this, the laboratory and diagnostic (LD) capacity of the faith-based healthcare facility where MVD outbreak was recorded in Ghana was significantly constrained. One of the participants recounted: “Infrastructure for the lab would also be appreciated. When a team came for research purposes, they were surprised that we could pick samples, store them, and transport them from such a small lab.” Others indicated that the space in the lab was very narrow that even two officers could not operate within it at the same time, hindering their capacity to meet the heightened LD demands during the outbreak. This observation runs parallel to the report by Africa CDC [9] confirming that under-resourced health facilities usually face logistical and operational difficulties during disease outbreaks [23]. Investing in infrastructural development would ensure quick and reliable diagnostics and reduce dependence on centralized testing centres [8].

Additionally, concerns were raised about extensive delays in sample transportation and feedback systems. The facility largely relied on external agencies, including the district disease control officers, for sample collection. One of them had indicated that the facility “kept the initial samples for 3 days before sending to Accra. In Accra too, it delayed for about two weeks before” feedback was received. This lag, which reflects the lack of investment in transportation infrastructure for FBOs, can hinder real-time clinical and public health decision-making and affect the integrity of samples taken [24]. Although the district disease control officers’ presence was commendable, showing excellent collaborative action against the outbreak, it also formed an additional layer to the coordination effort. When instructions are taken from different sources, it significantly slows down response time. According to the World Health Organization [8], health systems must have a reliable and decentralized sample transport system to reduce turnaround time for effective surveillance. It was observed that the facility could not autonomously dispatch samples due to rigid bureaucratic procedures. This practice deviates from WHO’s best practice [14] and undermines outbreak response efficiency. Given this, policies to decentralize sample transfer autonomy to competent FBOs during emergencies should be prioritised [8].

Turnaround time for diagnostic feedback was one of the dominant issues mentioned by the participants. As indicated, test results sometimes took over six days for feedback to be received. This is likely because the samples were first transported from the facility to Noguchi, and then to Dakar for confirmatory testing, before feedback was received. “Initially, results showed two positive cases… Later, the results came back as negative,” indicating discrepancies in diagnostic results. These discrepancies reduced the confidence of staff in diagnostic procedures, resulting in task neglects and untimely discharge of patients. It is worth mentioning that MVD is an RNA virus. RNA virus requires excellent cold chain to preserve it against possible degradation. Accordingly, given the infrastructural lags at the health facility, coupled with unsustainable financing of Noguchi, a false negative result may have occurred due to a breakdown in the cold chain during shipping. This finding highlights the criticalness of robust cold chain transport and storage conditions to preserve sample integrity. When test results delay, it could also impede contact tracing and public health containment efforts [14]. Contrary to global surveillance guidelines [8–10] which promotes shortened turnaround times for rapid isolation and containment, findings from this research reveals concerning gaps in the facility’s capacity for confirmatory tests. The reliance on international confirmation tests could also be the result of limited investment in lab and testing infrastructure or a lack of robust quality control systems for accurate diagnosis.

The need for reliable emergency response teams and contingency staffing plans to ensure operational integrity during crises is well established [14]. However, the study’s findings suggested that the surge capacity and coordination of workers were significantly strained. The facility isolated a significant number of staff due to exposure to MVD. This action was likely necessitated by the FBO’s rigid staffing structure, with most of its staff directly posted to the facility by the Ghana Health Service. Consequently, the remaining staff experienced burnouts, with no reserved manpower to handle the sudden increase in caseloads. According to Ashenafi, et al. [4], this deficiency can drastically reduce facilities’ capacity to test and diagnose.

On the positives, findings from the study show that biosafety protocols were well implemented. In particular, sample handling and staff protection was well secured. Participants indicated their strict adherence to cold chain logistics and PPE usage: “When transporting samples, we used a vaccine carrier with ice packs… we received proper training on how to put on and remove PPEs safely.” These accounts align with international standards that encourage biosafety to reduce healthcare-associated infections [23]. Despite this achievement, it was observed that since the facility was under-resource, the biosafety materials and training received may have been spearheaded or sponsored through donor supports. In other words, while the adherence reflects the potential of FBOs to ensure high biosafety standards when properly resourced and trained, the overreliance on external support could affect decision making and turnaround time during emergencies [23, 24].

Finally, the research explored the availability of testing kits and other protective gears during the outbreak. According to some of the participants, they did not have enough testing kits. Supply chain limitation has also affected the timely delivery of testing kits donated to the facility. Given that delayed testing for rare and highly sequenced pathogens may lead to the spread of the virus among workers, proper storage systems and PPE usage in emergency hotspots is indispensable. According to Ashenafi, et al. [4] delayed supply of critical LD equipment could hinder testing and diagnostic operations and heighten the occupational risk of staff. While acknowledging that the process of storing and dispatching samples was good, the FBO’s impaired capacity to perform independent tests due to logistical inadequacies does not urger well for disease control and surveillance efforts. This supply chain challenges were also stressed in the work of Marine, et al. [25] showing broader issues faced by LMICs, where unregulated procurement systems leave rural facilities without adequate supply of PPE and diagnostic kits during the disease outbreaks [23, 26].

## Limitations

The study has inherent limitations. Interviews were conducted one year post the MVD outbreak in Ghana, potentially leading to recall bias and forgetfulness. To mitigate this, we provided a brief background summary to each interviewee before posing substantive questions. Additionally, the study did not explore all eleven pillars of SPRP-AFR (2021), crucial for assessing emergency response and control capacity in health systems. Future investigations should therefore consider delving into these aspects. Given that the research was focused on participants’ interpretation of their outbreak experience at the facility level, national data on cold chain management, storage systems, and transportation of samples from Noguchi to Dakar could not be secured for analysis. Future research should adopt mixed-method approaches using data from government and other partner agencies in Africa to examine the standard checklists of the SPRP-AFR (2021).

## Conclusion

Well-coordinated LD is pivotal to effective health service delivery during emergencies. It ensures efficient resource allocation and planning for enhanced diagnostics and fosters coordinated efforts for timely and accurate testing. The findings of this research revealed that biosafety implementation was relatively strong. However, CHAG facilities are constrained by substantial barriers in infrastructure, transport logistics, turnaround times, and supply availability. These challenges are largely due to systemic gaps rooted in unfair resource allocation, rigid centralization, lack of emergency plans, and the neglect of FBOs in emergency planning. Given the challenges experienced in sample transportation, staffing, and storage, impacting the timeliness and accuracy of testing, CHAG must invest in enhancing staffing in labs, upgrading lab infrastructure, training the capacity of the officers, and promoting stronger collaboration with public health authorities for a quick response to future emergencies. A collaborative approach involving all key stakeholders, particularly religious organizations, the owners of CHAG facilities, and the government of Ghana, would strengthen the emergency preparedness and response capacities of faith-based healthcare institutions to better respond to public health emergencies in Ghana.

## Supplementary Information

Supplementary Material 1.

## Data Availability

The datasets used and/or analyzed during the current study are available and may be accessed from the corresponding author on reasonable request.

## Abbreviations

CHAG: Christian Health Association of Ghana
CMCT: Case Management, Clinical Operations, and Therapeutics
CPFM: Coordination, Planning, Financing, and Monitoring
EPR: Emergency Preparedness and Response
FBO: Faith-Based Health delivery Organizations
IPCP: Infection Prevention and Control and Protection of the Health Workforce
LD: Laboratories and Diagnostics
MVD: Marburg Virus Disease
OLS: Operational Support, Logistics, and Supply Chains
PIM: Points of entry, International travel, and transport, and Mass gatherings
RCCE-I: Risk Communication, Community Engagement, and Infodemic Management
RIE: Research, Innovation, and Evidence
SEHSS: Strengthening Essential Health Services and Systems
SOPHS: Surveillance, Outbreak Investigation, and Calibration of Public Health and Social Measures
SPRP: Strategic Preparedness and Response Plan
V: Vaccination
WHO: World Health Organization
