# Supplementary material for: Coordination of laboratory and diagnostic services during public health emergencies: a qualitative study
Source: BMC Public Health. 2026 Feb 5;26:824. doi: 10.1186/s12889-026-26422-4 (PMC12969918; doi:10.1186/s12889-026-26422-4)
Supplement: Supplementary file 1 — Supplementary Material 1. [file 12889_2026_26422_MOESM1_ESM.docx]

Appendix 2: Consolidated criteria for reporting qualitative studies (COREQ): 32-item checklist

Developed from: Tong A, Sainsbury P, Craig J. Consolidated criteria for reporting qualitative research (COREQ): a 32-item checklist for interviews and focus groups. International Journal for Quality in Health Care. 2007. Volume 19, Number 6: pp. 349 – 357

| **No. Item** | **Guide questions/description** | **Reported**  **on Page #** |
| --- | --- | --- |
| **Domain 1: Research team and reflexivity** | | |
| *Personal Characteristics* | | |
| 1. Interviewer | HNKA, GAO | 19 |
| 1. Credentials | All researchers comprised clinical practitioners, public health workers, and health service managers, engaged in core health management duties and in academia. | 1 |
| 1. Occupation | HANK (M.Phil) is a social worker and a doctoral candidate at the University of Ghana when interviews were conducted. The other researchers held clinician, faculty, and/or health service management positions. GAO (M.Phil) is a pharmacist and a doctoral student, GAA (M.A) is a health service manager and a doctoral student, and LY (Ph.D) is a professor of health service management. | 1, 27 |
| 1. Gender | Researchers comprised 2 males and 2 females. | 1 |
| 1. Experience and training | All researchers have extensive experience in various healthcare areas, including public health and mental wellness, health facility management, and clinical care services. All researchers have experience with both quantitative and qualitative research methods. | 1 |
| *Relationship with participants* | | |
| 1. Relationship established | The interviewers were not directly engaged with the research facility or the participants. | 7-8 |
| 1. Participant's knowledge of the interviewer | The interviewers introduced themselves to the participants and explained their role as research officers. They explained that all interviews would be de-identified and that findings would be reported only in aggregate form. The study’s purpose, to explore participants’ initial impressions and perceived preparedness of faith-based facilities against emergencies, was clearly communicated. Internet data support was provided to participants only upon request. | 7-8 |
| 1. Interviewer characteristics | The interview team consisted of a health research officer/social worker and a pharmacist affiliated with CHAG; however, neither was directly working with the facility nor any of the research participants. | 1, 7-8 |
| **Domain 2: study design** | | |
| *Theoretical framework* | | |
| 1. Methodological Orientation and Theory | The study adopted the explanatory case study design, informed by the WHO Coronavirus Disease 2019 (COVID-19) Strategic Preparedness and Response Plan for the African Region (SPRP-AFR, 1 February 2021–31 January 2022). The qualitative research method was used. | 6-8 |
| *Participant selection* | | |
| 1. Sampling | Non-probability, purposive sampling | 7 |
| 1. Method of approach | Zoom | 7 |
| 1. Sample size | 15 | 7, 9 |
| 1. Non-participation | A total of 25 participants, including 7 nurses and 8 enrolled nurses, were invited to participate in interviews. Although all invited participants responded favorably, saturation was reached after the 15th interview. | 7, 9 |
| *Setting* | | |
| 1. Setting of data collection | All participants were drawn from the district hospital where the MVD was identified. Because the interviews were conducted through video-based communication platforms, participants’ physical locations at the time of the interviews were not determined. | 7 |
| 1. Presence of non- participants | Only the participant and the two interviewers were present at each interview. | 7 |
| 1. Description of sample | Both clinical and non-clinical health workers who were employees of the district hospital where the study was carried out were interviewed. | 7, 9 |
| *Data collection* | | |
| 1. Interview guide | Semi-structured, tested with the study team through 3 pilot interviews. | 7 |
| 1. Repeat interviews | No | 7 |
| 1. Audio/visual recording | Audio recording | 7 |
| 1. Field notes | No. | 7 |
| 1. Duration | 20 to 45 minutes | 7 |
| 1. Data saturation | Yes | 7 |
| 1. Transcripts returned | No | 7 |
| **Domain 3: analysis and findings** | | |
| *Data analysis* | | |
| 1. Number of data coders | 4 | 27 |
| 1. Description of the coding tree | Data analysis followed a three-stage coding process. First, open coding was used to label meaningful segments of interview transcripts. Second, axial coding grouped related codes into categories by identifying patterns and relationships. Finally, selective coding integrated core categories to develop overarching themes that explained participants’ shared experiences. | 7 |
| 1. Derivation of themes | Inductive approach. | 7-9, 22 |
| 1. Software | NVivo | 7-8 |
| 1. Participant checking | 3 | 8 |
| *Reporting* | | |
| 1. Quotations presented | Yes, quotations are presented in a de-identified fashion. | 9 - 20 |
| 1. Data and findings consistent | Consistency between the data and the findings exists. | 10-24,  22-26 |
| 1. Clarity of major themes | Major themes are clearly identified and described. | 5-6, 9, 28 |
| 1. Clarity of minor themes | Minor themes are clearly identified and described. | 5-6, 9, 28 |
